# Defective Phagocytic Properties of HIV-Infected Macrophages: How Might They Be Implicated in the Development of Invasive *Salmonella* Typhimurium?

**DOI:** 10.3389/fimmu.2018.00531

**Published:** 2018-03-23

**Authors:** Gabrielle Lê-Bury, Florence Niedergang

**Affiliations:** ^1^INSERM, U1016, Institut Cochin, Paris, France; ^2^CNRS, UMR 8104, Paris, France; ^3^Université Paris Descartes, Sorbonne Paris Cité, Paris, France

**Keywords:** macrophages, HIV, *Salmonella*, phagocytosis, opportunistic diseases

## Abstract

Human immunodeficiency virus type 1 (HIV-1) infects and kills T cells, profoundly damaging the host-specific immune response. The virus also integrates into memory T cells and long-lived macrophages, establishing chronic infections. HIV-1 infection impairs the functions of macrophages both *in vivo* and *in vitro*, which contributes to the development of opportunistic diseases. Non-typhoidal *Salmonella enterica* serovar Typhimurium has been identified as the most common cause of bacterial bloodstream infections in HIV-infected adults. In this review, we report how the functions of macrophages are impaired post HIV infection; introduce what makes invasive *Salmonella* Typhimurium specific for its pathogenesis; and finally, we discuss why these bacteria may be particularly adapted to the HIV-infected host.

## Clearance and Activation Capacity of Macrophages

Although macrophages are different in the different tissues and organs in which they reside, they can all be characterized by their strong capacity to internalize and degrade particles in phagolysosomes. Transcriptomic analysis has pointed to unique gene expression profiles in response to pathogens components (1). One obvious functional set of genes shared by all phagocytes encodes for the components of lysosomes, such as the vacuolar ATPase H^+^ pump and lysosomal hydrolases. Subtle differences can be revealed when comparing the clearance capacities of polymorphonuclear neutrophils, macrophages or dendritic cells. Clearance is the hallmark of neutrophils and macrophages, while dendritic cells have been reported to be milder with internalized material, with some material remaining undegraded (2–5). In this review, we will focus on macrophages, as they are target cells for both the human immunodeficiency virus type 1 (HIV-1) and *Salmonella* pathogens.

Phagocytosis begins with the clustering of receptors that are engaged by ligands present on the surface of the target particle. Many types of receptors can be implicated in the recognition step, regulating the fate of the internalized material. Phagocytic receptors can be subdivided into receptors that bind to opsonins, like the immunoglobulins and complement, and receptors that bind to non-opsonins. The latter interact with molecular groups on the surface of the target particle or pathogen, including sugars, lipids, and polypeptides that are referred to as pathogen-associated molecular patterns (6, 7). Early signaling from surface receptors leads to the polymerization of actin that drives plasma membrane deformation and the formation of a closed phagosome (8, 9). For large targets to be efficiently internalized, membrane remodeling is essential, relying on the focal delivery of intracellular compartments including recycling endosomes (10–12). The large GTPase dynamin2 is crucial for phagosome sealing (13).

Once closed, the phagosome evolves very much like smaller endocytic compartments, undergoing a series of fusion and fission events with the compartments of the endocytic pathway (14, 15). This process, called phagosome maturation, is accompanied by the dynein-mediated movement of the phagosome along microtubules (16, 17). The microtubule network is crucial for the phagosome to reach the lysosomes positioned at the center of the cell and to efficiently mature into a phagolysosome. Indeed, it has been demonstrated that the loading of dyneins at the plus ends of microtubules by the plus-end binding protein, EB1, is critical for phagosome maturation (18).

Early phagosomes harbor markers of the early endosomes such as Rab5 and its effector, the early endosome antigen 1 (EEA1) (19). Other effectors of Rab5 are the class III phosphatidylinositol 3 kinase human vacuolar protein-sorting 34 that generates phosphatidylinositol 3-phosphate [PI(3)P] (20). It has been demonstrated that PI(3)P is important after phagosome completion, for its maturation (21). EEA1 carries a FYVE domain that binds to PI(3)P, a zinc finger that binds to Rab5 and regions responsible for multimerization. EEA1 also binds to the t-SNARE Syntaxin-13, which is important for membrane fusion (22). Rab5 exchange factors, including Rabex-5, Rin1, and Gapex-5, coordinate Rab5 activation and microtubule dynamics (23).

Acquisition of Rab7 is still considered to be a hallmark of late phagosomes, although a choreography of Rab proteins has been shown to be recruited during phagocytosis (24). The products of PI3K are involved in the dissociation of Rab5, but are not essential for the recruitment of Rab7 on the phagosome (25). Data in yeast have shown that the proteins Mon1 and Ccz1 serve as a Rab7 exchange factor (26). Phagosomes undergo fusion with late endosomes and lysosomes *via* a Soluble NSF attachment protein receptor (SNARE) mediated process. It has been demonstrated that Syntaxin 7 and Syntaxin 8, with VAMP7 and VAMP8, are involved in phagosome-lysosome fusion (27). The vpsC–homotypic protein sorting (HOPS) complex that mediates the transition from Rab5 to Rab7 endosomes could play a similar function in phagosome maturation. The complex is composed of Vps11, Vps16, Vps18, Vps33, Vps39, and Vps41. In yeast, Rab7 is activated by Vps39. The Vps41 protein is a key component of the HOPS complex as it is required for the stabilization of the HOPS complex on the endosomal membrane before fusion with the vacuole. Regulation by the p38 MAP kinases of Vps41 has recently been highlighted as an explanation for the differential trafficking of virulent LPS of *Coxiella burnetii* (28). This type of regulation might also be implicated in phagosome maturation in macrophages. Rab7 and Arl8 orchestrate both microtubule-dependent transport of late endosomes/lysosomes and their fusion with endosomes, autophagosomes, and phagosomes. Both proteins are important for lysosome tubulation in macrophages (29). Rab7-interacting lysosomal protein and the long splice-variant of oxysterol-binding protein related-protein 1 (ORP1L) function together to link phagosomes to the microtubule motor dynein/dynactin (30, 31). Arl8 has been shown to control phagosome maturation and bacterial killing (32). It has also been demonstrated that Arl8 plays an important role in phagosome maturation in *Caenorhabditis elegans* (33). Arl8 connects lysosomes to kinesins through SifA and kinesin interacting protein (34), in particular, to kinesins 1 and 3, controlling lysosomal positioning (35). Some effectors, like PLEKHM1, act as dual effectors for Rab7 and Arl8 to promote cargo delivery to lysosomes (36) and may also play a role during phagosome maturation.

Acidification occurs gradually while the compartment matures into a phagolysosome, which is important for the optimal activity of hydrolytic enzymes delivered by late endosomes and lysosomes. Acidification reaches a pH of 5.5 in the late phagosome, due to the acquisition of proton pumping vATPases. Negatively charged chloride ions may enter the compartments to compensate for the proton influx, although this role has not been established experimentally. However, the depletion of luminal cations, Na^+^ and K^+^, during maturation has been established (37). Anion and luminal cation exchange may serve to maintain lysosome osmolarity and volume during the acidification steps.

Microbicidal activity in the phagolysosome depends both on hydrolases and on the generation of reactive oxygen and nitrogen species (ROS and RNS, respectively) (38). The NADPH oxidase is acquired at the early stages of phagosome maturation. Its activity is complemented by the inducible NO synthase, iron scavengers, and transporters, as well as lysozymes, lipases and proteases, such as the cathepsins. These species are delivered to phagosomes and directly contribute to killing. Their activities can be modulated by cell activation and the concomitant signaling pathways that are initiated downstream of surface receptors in complex regulatory loops. For instance, the interaction between the RUN domain Beclin-1 interacting cysteine-rich-containing (RUBICON) protein and the NADPH oxidase upon TLR stimulation forms a feedback loop with the cascade signaling to cytokine production (39).

Finally, when degradation is incomplete, antigens derived from the internalized material are presented on the major histocompatibility complex molecules (Class I or II), especially in dendritic cells. Signaling pathways are activated and the phagosome itself can be considered a signaling platform. Together, these events lead to the production of cytokines and inflammatory mediators, which can be modulated depending on the surface receptors engaged by the cargo (40, 41).

## Dysregulation of Macrophage Phagocytosis by HIV Infection

The initial detection of the acquired immunodeficiency syndrome (AIDS) epidemic in 1981 began with reports of an unusual syndrome in which previously healthy young males were presenting with diseases such as Kaposi’s sarcoma, cytomegalovirus pneumonia, and *Pneumocystis carinii* pneumonia, previously described in immunocompromised patients (42). The syndrome was then named AIDS. Indeed, the principal effect of the virus is to decrease the immune defenses of infected individuals, leading to the appearance of opportunistic diseases and tumors. Invasive pneumococcal and oral candidosis presented early in HIV-infected patients, especially in Africa, together with non-typhoidal *Salmonella* infections, which will be the focus of the following section in this review.

The introduction of highly active antiretroviral therapy (HAART) has radically altered the incidence of AIDS, initially in developed countries, and later on, worldwide. However, even with HAART, which results in undetectable plasma HIV loads, HIV transcription persists in reservoirs, such as long-lived memory T cells, phagocytic cells, as well as other cell types in various tissues (43).

Already in the 1980s, Crowe et al. reported that macrophages are targets for HIV-1 and may act as major reservoirs of virus (44, 45). It has since been established that there is some specificity in the infection cycle of HIV-1 in macrophages (46–49) and see Ref. (50) for a recent review in the same topic.

**Figure 1:**
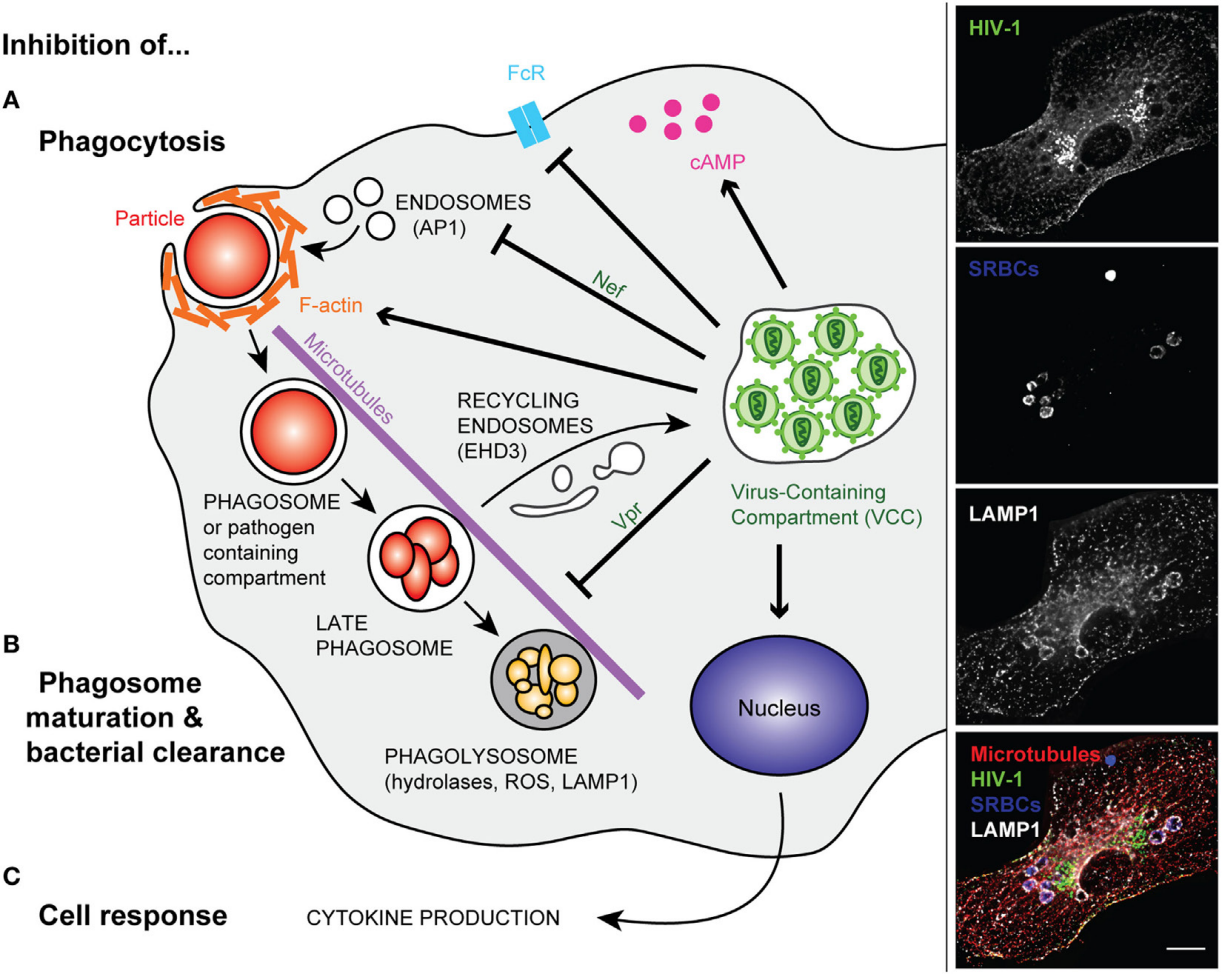
Modified phagocytosis in HIV-infected macrophages. Left panel—Once HIV infection of a macrophage is established, intracellular trafficking is rerouted to the virus-containing compartment (VCC). In non-infected macrophages, phagocytosis is initiated by the binding of phagocyte surface receptors to ligands present on the microorganism or to the opsonizing molecules that coat the target particle. In HIV-infected cells, surface receptors (e.g., FcR) can be downregulated **(A)**. After binding, a cascade of signaling events leads to actin polymerization and engulfment of the particulate material in a closed compartment termed, the phagosome. The inhibition of phagocytosis in HIV-infected macrophages was related to perturbation of F-actin and cAMP production. The viral factor Nef further reduces the efficiency of phagosome formation *via* its interaction with the AP1 adaptor protein, reducing the focal delivery of intracellular compartments **(A)**. In non-infected macrophages, the phagosome matures into a degradative compartment called phagolysosome. This occurs after fusion and fission with various endocytic compartments, and the phagolysosome migrates along microtubules. However, in HIV-infected macrophages, the viral factor Vpr inhibits phagosome maturation and centripetal movement of the phagolysosome toward the nucleus. Part of the intracellular trafficking, such as the EHD3 recycling machinery, is rerouted to the VCC **(B)**. In addition, viral infection inhibits macrophage late events and responses such as cytokine production **(C)**. Right panel—Primary human macrophages were infected with HIV-1_ADA_ for 8 days before incubation with IgG-opsonized sheep red blood cells (SRBCs) for 60 min at 37°C. They were fixed, permeabilized, and labeled with anti-p24 followed by Alexa488-anti-goat IgG (upper line), AMCA-anti-rabbit IgG to detect the total SRBCs (second line), anti-LAMP1 followed by Cy3-anti-mouse IgG (third line), and anti-tubulin followed by Cy5-anti-human IgG (not shown). Merged images (lower line) show p24 in green, SRBCs in blue, LAMP1 in white, and microtubules in red. Z stacks of wide field fluorescent images were acquired, deconvoluted, and treated with ImageJ. Bar, 10 µm.

The group of Crowe has performed pioneering work in analyzing the phagocytic capacity of blood monocytes from a small sample of the Sydney Blood Bank Cohort or *in vitro* differentiated and infected macrophages. They studied phagocytosis of apoptotic neutrophils, a *Mycobacterium avium* complex, *Candida albicans, Toxoplasma gondii*, and IgG- or complement-opsonized targets (51–55). The data suggested that phagocytosis by monocytes from WT HIV-1-infected individuals was impaired, whereas the phagocytic capacity of the phagocytes from the Δ*nef* HIV-1-infected subjects was not. Interestingly, no correlation was found between the level of inhibition of phagocytosis and the viral load or the CD4 counts (52). The authors reported an increased level of basal F-actin in HIV-infected cells, which could account for a defective capacity of the actin cytoskeleton to efficiently remodel during phagosome formation (Figure 1A). Some HIV-1 induced receptor downregulation was observed in primary human macrophages (56), as in alveolar macrophages for mannose receptors (57). The group of Crowe also pointed to a crucial role of the Nef viral protein in impairing phagocytosis in infected blood monocytes *in vivo*, a phenotype that they did not observe in monocyte-derived macrophages-infected *in vitro*. Nef was reported to downregulate many surface receptors and markers from the surface of treated model cells (58, 59), as well as CD36 on macrophages, which may be related to defective phagocytosis of inert particles and killed bacteria in Nef-treated cells (60). Other reports showed that HIV-1 infection did not induce any decrease in the surface expression of phagocytic receptors (52, 61). The impact of *in vitro* infection of macrophages with HIV-1 on various types of phagocytosis was analyzed. The focal delivery of endosomal compartments at the site of phagocytosis was impaired in a Nef-dependent manner, probably due to Nef interaction with the adaptor complex, AP1, on endosomes (61) (Figure 1A). This endosomal delivery was shown to be required for efficient phagocytosis of large particulate material (11, 62), and interestingly, to be controlled by the NF-κB signaling protein Bcl10 (63). Other molecular defects reported in HIV-infected macrophages include elevated intracellular cAMP levels (64) and decreased expression of the common gamma chain (65) (Figure 1). Of particular significance, defective phagocytosis was also reported in the population of small alveolar macrophages in the lung of HIV-infected patients (66).

The later steps of phagosome maturation were also reported to be impaired in HIV-infected macrophages, with early reports showing that the intracellular replication of live *T. gondii* was enhanced in HIV-infected macrophages. Interestingly, treatment of macrophages with interferon gamma, a known activator of these cells, decreased parasite replication, but did not control parasite levels (53). An assay developed in the laboratory of Russell to monitor the superoxide burst in phagocytes by flow cytometry was used to analyze whole blood samples of HIV/tuberculosis-infected individuals (67). The authors were able to demonstrate an impaired superoxide burst activity in the phagocytes of coinfected patients. Using similar tools together with other assays, it was determined that Nef was not crucial to the *in vitro* mediation of the phagosome maturation defect in HIV-infected macrophages, while, unexpectedly, the viral protein Vpr, was (Figure 1B) (18). Vpr perturbs the microtubule dynamics, the localization of the plus-end microtubule binding protein EB1 and therefore, the positioning of the dynein motors necessary for driving phagosomes to the cell center. In addition, the viral infection of macrophages relies on the budding of newly enveloped viral particles in virus-containing compartments (VCCs), which presumably requires the recruitment of large amounts of membrane. Part of the intracellular endocytic machineries are de-routed toward the VCCs in HIV-1-infected macrophages, as is the case for the EHD3 sorting protein (18). It is therefore probable that many of the intracellular trafficking pathways are altered in virus-infected cells, as a side effect of viral particle production. This may benefit many opportunistic pathogens in a non-specific manner.

Similarly, signaling to initiate cell activation and cytokine production is altered in HIV-infected cells (Figure 1C). Placental blood mononuclear cells purified from HIV-infected mothers constitutively secrete more IL-1β and IL-6 and have more IL6, IL1β, and TNF-α mRNA; however, the high basal rates of secretion were associated with a lower response to stimulation with LPS (68). Reduced cytokine production was also observed in HIV-infected macrophages that were triggered for receptor-mediated phagocytosis or infected with bacteria (18). Many pro-inflammatory cytokine signaling pathways rely on the activation of the NF-κB pathway. The latter is transiently activated during the activation of transcription *via* the HIV promoter or the long terminal repeats. This may result in inadequate subsequent activation when the cells are subjected to a secondary trigger. Reduced intracellular protein levels of FcRγ, the signaling adaptor protein and chaperone required for FcγRI and III expression and function, were reported (69). Inhibition of subsequent downstream phosphorylation of Hck and Syk tyrosine kinases was observed in HIV-infected monocyte-derived macrophages undergoing Fcγ receptor-mediated phagocytosis.

## Unique Intrinsic Properties of Invasive Non-Typhoidal Salmonellae (iNTS)

In as early as 1990, non-typhoidal salmonellae (NTS) were confirmed as HIV-related pathogens in sub-Saharan African adults (70). Later, NTS bacteremia has become a common and recurrent illness among susceptible African children and HIV-infected adults (71). This bloodstream infection, in African, HIV-infected adults, was reported to have high mortality (47%) and recurrence (43%) rates (72), due to recrudescence and reinfection (73). The bacteremia may be due to both *Salmonella enterica* serovar Typhimurium and *Salmonella enterica* serovar Enteritidis (74).

*Salmonella enterica* is a Gram-negative bacterium that causes enteric diseases. The species, *S. enterica*, includes typhoidal and non-typhoidal *Salmonella* and comprises a large number of serovars. *S. enterica* serovars Typhi and Paratyphi cause typhoid and paratyphoid fevers, respectively. The pathogens penetrate through the intestinal mucosa, producing bacteremia and lodge in the macrophages of the reticuloendothelial system. The remaining serovars normally lead to a self-limiting diarrheal disease in healthy individuals, but some NTS, such as *Salmonella* Typhimurium, can cause bloodstream infection in immunocompromised adults (75). Thus, iNTS have emerged as a prominent cause of bacteremia in African individuals with an associated case fatality of 20–25% (76).

Multilocus sequence typing (MLST) analysis of numerous isolates of *Salmonella* Typhimurium from Malawi and Kenya revealed new sequence type variants of *S*. Typhimurium associated with iNTS in sub-Saharan Africa. This dominant regional genotype, MLST group ST313, presents several genetic differences compared with other strains of this serotype, such as NTS ST19 (77).

In addition, whole-genome sequence-based phylogenetic methods revealed that the majority of ST313 isolates fell within two closely related, clustered phylogenetic lineages: lineage I, with A130 as a hallmark strain; and lineage II, with D23580 as a hallmark strain (78). These lineages are distinct from other *S*. Typhimurium lineages due to their distinct metabolic profiles (79) and antibiotic resistance (77). Indeed, Okoro et al. observed that isolates from lineage II appeared after the use of chloramphenicol for the treatment of iNTS disease, suggesting a clonal replacement of isolates from lineage I, by those from lineage II influenced by antibiotic usage. In addition, the authors estimated that lineage I and lineage II appeared independently, ~52 and ~35 years ago, respectively, and then developed with the HIV pandemic. Thus, Okoro et al. propose that iNTS disease, in sub-Saharan Africa, is caused by highly related *Salmonella* Typhimurium lineages that may have developed in immunosuppressed populations and following antibiotic treatment (78).

Host-adapted *Salmonella* serovars that cause invasive disease such as *S*. *enterica* Typhi and *S. enterica* Paratyphi display some similarities to ST313 isolates, in particular some genome degradation (80). Compared with non-iNTS *S*. Typhimurium isolates, ST313 isolates present numerous pseudogenes and deletions (77). In addition, whole-genome comparisons of a representative isolate of ST313, D23580 from Malawi, and ST19 strains (LT2, SL1344, and DT104) revealed a distinct repertoire of six prophage-like elements. These include five full-length prophages arbitrarily named Blantyre Prophage “BTP” 1 through 5, and one prophage remnant (77). Okoro et al. highlighted that this set of prophage sequences is present in all ST313 isolates belonging to lineage I and II (79). Among the five full-length prophages, three of them were already well-characterized, and commonly found in *S*. Typhimurium genomes: Gifsy-2^D23580^ (BTP2), ST64B^D23580^ (BTP3), and Gifsy-1^D23580^ (BTP4) that are all defective in ST313. The two remaining, BTP1 and BTP5, are novel prophages that are found only in the ST313 genome. BTP1 contains three virulence-related genes: *st313-td, gtrCc*, and *gtrAc* (81). *st313-td* was reported to play a role in survival within murine macrophages and in virulence in a mouse model of bacterial infection (82). The *gtrAC* operon encodes an *O*-glycosyltransferase that modifies the composition and the length of O-antigen of the bacterial lipopolysaccharide (83). Further, the LPS of D23580 (ST313) present particular O-polysaccharide chains (84), which have been used to design glycoconjugate vaccines against invasive African *S. enterica* serovar Typhimurium (85, 86).

The D23580 isolate has four plasmids including one virulence-associated plasmid. This plasmid contains an insertion that resembles a composite *Tn21*-like mobile element encoding multiple drug resistance genes (77). Before the appearance of MDR *S*. Typhimurium, ST313 strains of lineage I were susceptible to chloramphenicol. The selection of this virulence-associated plasmid explains the emergence of MDR *S*. Typhimurium, such as D23580 (lineage II), associated with the epidemic increase in the incidence of iNTS in Malawi after chloramphenicol treatment (74).

After an analysis of 129 ST313 isolates, Yang et al. demonstrated that these exhibit a distinct metabolic signature compared with non-ST313 *S*. Typhimurium. For instance, D23580 seems to be more resistant to acid stress than non-ST313 *S*. Typhimurium (87). Among the differences in metabolic pathways, ST313 strains present two loss-of-function mutations that impair multicellular stress resistance associated with survival outside the host. Hence, ST313 bacteria are less resistant to oxidative stress than ST19 (87, 88), due to mutations causing inactivation of KatE catalase in ST313. Catalase converts hydrogen peroxide (H_2_O_2_) to oxygen and water and this H_2_O_2_ detoxification protects high-density bacterial communities from oxidative stress. Another loss-of-function mutation in the *bcsG* gene in D23580 induces an inactivation of the BcsG cellulose biosynthetic enzyme required for the RDAR (red, dry, and rough) colonial phenotype (88). RDAR colonies represent a form of multicellular behavior that enhances *Salmonella* stress resistance in the environment and allows biofilm formation (89). A comparative analysis of biofilm-forming ability and long-term survival has shown that ST19 strains, that are strong biofilm producers, can survive desiccation better than ST313 that form weak biofilms and survive poorly following desiccation (90). In addition, several ST313 strains express less flagellin, a component of the flagellum appendage responsible for bacterial motility (91, 92). Indeed, ST313 strains (D65, Q55, S11, S12, D23580, and A130) were reported to be less motile than ST19 strains (I77, I89, I41, S52, and SL1344) (91, 93), although some variability among the strains was observed (87). These data suggest that, like *Salmonella* Typhi, *Salmonella* Typhimurium ST313 lack some of the mechanisms that allow transmission and/or survival in the environment.

## *Salmonella* Typhimurium and Host Interactions

*Salmonella* Typhimurium were reported to be quickly taken up by CD18^+^ cells in the blood after oral infection (94). *Salmonella* Typhimurium are also taken up by CCR6^+^ phagocytes located in the subepithelium dome of Peyer’s patches in the intestine, then carried to the mesenteric lymph node (95). In addition, dendritic cells were shown to be able to extend dendrites toward the intestinal lumen, without perturbing the tight junctions of epithelial cells, to capture bacteria (96). *Salmonella* Typhimurium persist within macrophages in the mesenteric lymph nodes of chronically infected Nramp1 (natural resistance associated macrophage protein 1)^+/+^ mice (97). *S*. Typhimurium may be killed by the cell (98) or can hijack the host cell defenses to survive inside the cell (99–101). More recently, it has been recognized that macrophage polarization can influence bacterial infection. A macrophage population is heterogeneous in its susceptibility to the infection, potentially due to a mixture of type 1 and type 2 macrophages, as shown *in vitro* with mouse bone marrow-derived macrophages (102). *S*. Typhimurium cannot replicate in primary human monocyte-derived macrophages polarized into inflammatory M1 macrophages, while M2 and M0 macrophages allowed bacterial replication (103). The tissue source of macrophages further determines the degree of growth or survival of bacteria. For example, *S*. Typhimurium seems to survive better in splenic macrophages than in peritoneal macrophages (104). This is consistent with the main reported site of *Salmonella* infection (105).

At the cellular level, *S*. Typhimurium can enter in macrophages by SPI1 (*Salmonella* pathogenicity island-1)-dependent invasion (106) or by host cell-mediated phagocytosis or macropinocytosis, which occurs through either SPI-1-dependent or SPI-1-independent mechanisms (107). After invasion, *S*. Typhimurium resides in a spacious phagosomal compartment that evolves to form a specific compartment called *Salmonella*-containing vacuole (SCV) (108). It is important to note that *Salmonella* finely regulate virulence gene expression while replicating inside a macrophage (109). Thus, bacteria can survive within the SCV, despite partial fusion with the lysosomal compartment (110). Acidification of the compartment induces the transcription of virulence genes of *S*. Typhimurium to inhibit macrophage phagosome acidification (111). In addition, the *Salmonella* pathogenicity island-2 encodes proteins required for bacterial replication (112, 113) but does not have a major influence on resistance to killing (114). Intracellular bacteria can exhibit large heterogeneity in growth rate inside the vacuolar environment of host cells. A segment of the bacterial population does not replicate inside the cell but instead appears to enter a dormant-like state, perhaps providing a reservoir for relapsing infection (114, 115). Recent transcriptomic analysis demonstrated that macrophages containing non-growing bacteria are in a pro-inflammatory state of polarization. By contrast, macrophages containing growing bacteria exist in an anti-inflammatory, M2-like state. Thus, the growth arrest of *Salmonella* seems to facilitate immune evasion and the establishment of a long-term niche, while macrophages with replicating bacteria allow *Salmonella* to escape intracellular antimicrobial activity and proliferate (116). The heterogeneous activity of bacterial factors in individual infecting bacteria determines the heterogeneity of immune responses of individual-infected host cells (117). Macrophage infection induces the production of pro-inflammatory mediators (118) but also leads to macrophage cell death (119), an essential virulence mechanism of *Salmonella* Typhimurium (120). One form of cell death, pyroptosis, occurs either *via* a rapid caspase-1-mediated SPI-1-dependent pathway or a delayed SPI-2-dependent caspase-1-mediated pathway. Caspase-1, a central effector of pyroptosis, is activated in the inflammasome complex during *Salmonella* infection and has a protective role during *Salmonella* infection *in vivo* (121).

## Host–Pathogen Interaction of iNTS

**Figure 2:**
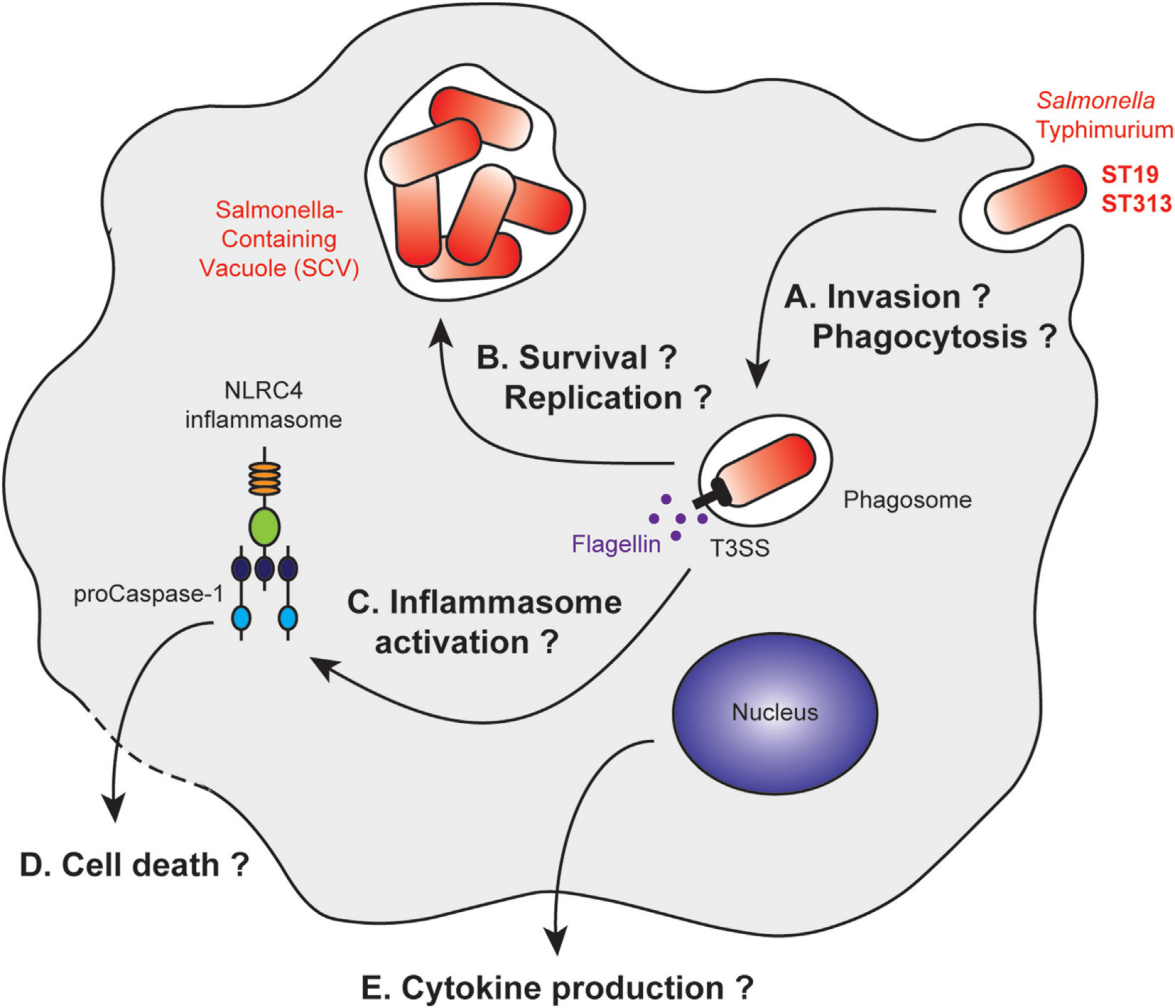
Stages of *Salmonella* Typhimurium infection with differential macrophage response to ST19 or ST313 infection. After macrophage invasion **(A)**, *Salmonella* Typhimurium resides in vacuoles called *Salmonella*-containing vacuoles (SCV). The bacteria form this vacuole to prevent maturation of the phagosome into a phagolysosome **(B)**. Bacterial products, such as flagellin, are released after membrane rupture, or secreted into the cytosol. This leads to inflammasome activation **(C)** and IL1β production, which are associated or not with cell death pathways **(D)**. Other cytokines are released by the macrophage, and their secretions could be differentially regulated **(E)**.

In several studies, gentamicin protection assays were used to assess the invasion properties of the ST19 and ST313 strains *in vitro* (Figure 2A). Two studies found that ST313 invades less Hep2 and HeLa cells than ST19 (79, 92). By contrast, Herrero-Fresno et al. observed no difference in invasion of another human cell line (human epithelial Int407 cells) with ST19 (4/74) and ST313 (02-03/002) (82). In the final study, ST313 (D23580) was shown to be more invasive than ST19 (14028) in HeLa cells (88). Studies pertaining to macrophages have also generated conflicting data. J774 mouse macrophages phagocytose ST313 (D65, Q55, S11, S12, and A13) more efficiently than ST19 (I77, I41, S52, and I89) (91). Bone marrow-derived C57BL/6 macrophages are highly phagocytic of both ST19 (SL1344 and DT104) and ST313 (D23580, A130, 5597, and 5579) (92). Taken together, these results do not indicate major differences in the invasive capacity of the two strains.

The intracellular survival of the ST19 and ST313 strains was analyzed in various model systems, resulting in variable and contradictory data (Figure 2B). Ramachandran et al. demonstrated that ST313 (D65, Q55, S11, S12, and A13) survives better than ST19 (I77, I41, S52, and I89) within macrophages using several cell lines and primary cells (U937 cells, THP-1 cells, peritoneal macrophages from BALB/c mice and CD-1 mice, human peripheral blood mononuclear cells) (91). Two other studies in murine cell lines (J774 mouse macrophages, RAW264.7 murine macrophages-like cells), however, have shown that there are no differences in survival between the two strains (82, 88). These differences could be due to the opsonization of the bacteria, their growth phase at the time of infection, and the degradative properties of the infected cells (82, 88, 91). To date, no precise characterization of the SCV has been performed to understand whether there is a difference in the intracellular survivability of two strains.

Initially, it was observed that both strains, ST19 (4/74) and ST313 (02-03/002) induce the same cytotoxicity toward J774 mouse macrophages (82). By contrast, two studies on bone marrow-derived C57BL/6 macrophages (92) and THP-1 cells (91) have shown that ST313 (D65, D23580, A130, 5597, and 5579) induces less macrophage death than ST19 (I77, SL1344, and DT104). Cytotoxicity appears to be dependent on the NLRC4 inflammasome (92). However, a more recent study has suggested that ST313 (D23580) is more cytotoxic than ST19 (14028) toward the RAW264.7 murine macrophage cell line (88) (Figures 2C,D).

Cell activation was also compared in terms of cytokine production. ST313 (D65, D23580, A130, 5597, and 5579) induced less cytokine production by macrophages compared with ST19 (I77, SL1344, and DT104) for IL1β (91, 92), IL8, and TNFα (91). These studies were performed with the human THP-1 (91) and U937 cell lines (92), as well as with bone marrow-derived C57BL/6 macrophages (92) (Figure 2E).

To better understand NTS pathogenesis, different animal models can be used. The non-human primates, such as rhesus macaques, are especially useful for investigating coinfection with simian immunodeficiency virus (122). Calves can be used as infection models as their infection with *S*. Typhimurium results in a pathology similar to humans. Furthermore, *S*. Typhimurium is a natural pathogen of cattle, and beef is a common reservoir for human infection (123, 124). Poultry products are well known as a source of human infections (125). Interestingly, ST313 was reported to be more invasive than ST19 in experimentally infected chicken (126). Mice are not typically suitable as an adapted model for S. Typhimurium, as these bacteria induce a typhoid-like systemic illness that leads to death of the animals. Although the 50% lethal dose (LD_50_) was not reported to be significantly different between ST313- and ST19-infected mice (87, 93). Further, there was no consistent difference in the inflammatory response (79, 88, 93, 126) or in the ability of the bacteria to colonize the intestinal tract and to disseminate in the body (79, 87, 88, 92, 93, 126).

## What is Known about the Coinfections with HIV and *Salmonella*

**Figure 3:**
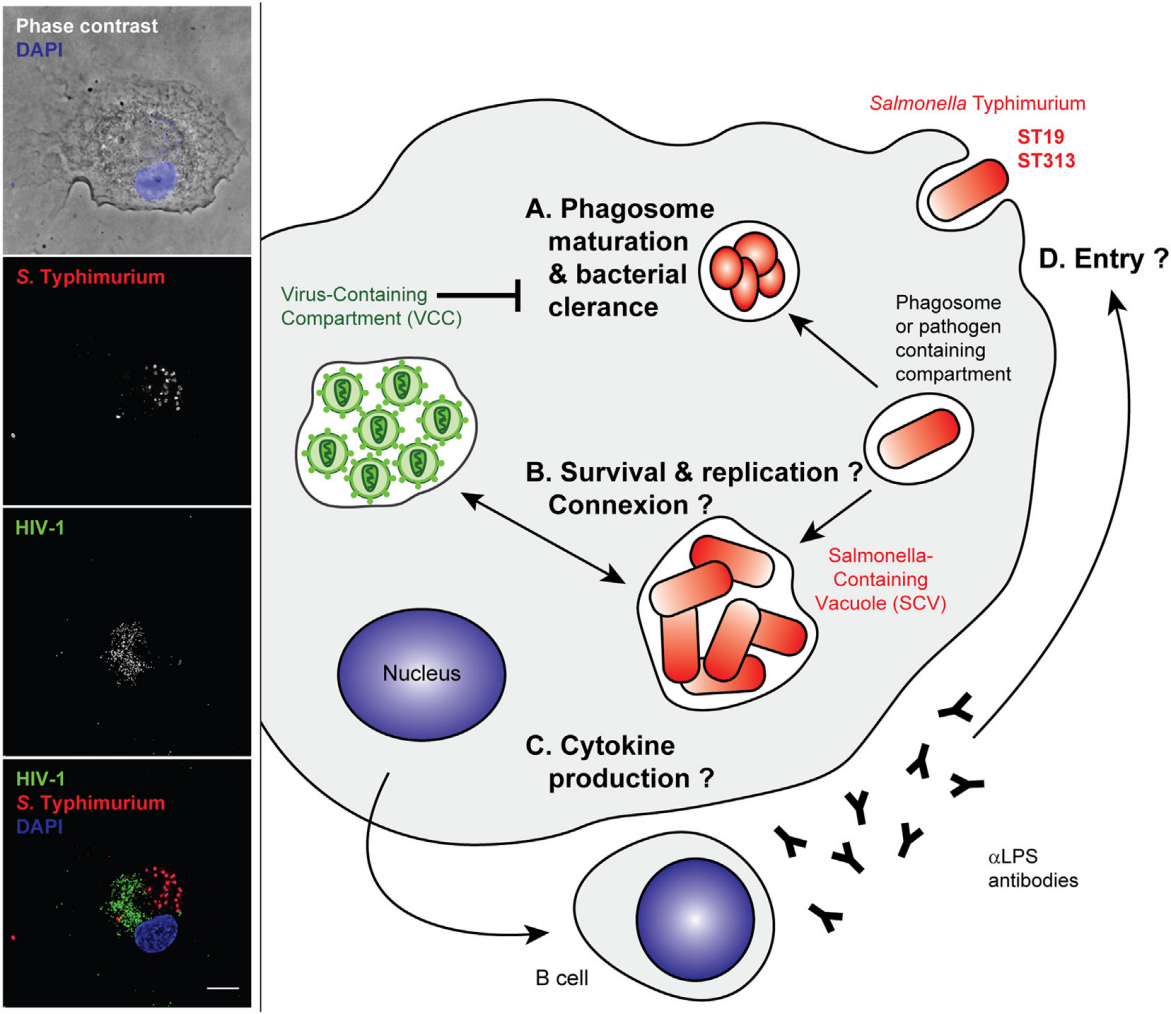
Stages of *Salmonella* Typhimurium infection that may benefit from an established human immunodeficiency virus type 1 (HIV-1)-infection of the host cell. Left panel—Primary human macrophages were infected with HIV-1_ADA_ for 8 days before incubation with invasive *Salmonella* Typhimurium for 6 h at 37°C. They were fixed, permeabilized, and labeled with anti-p24 followed by Alexa488-anti-goat IgG (third line), anti-LPS followed by Cy5-anti-rabbit IgG (second line), and DAPI. Phase contrast image with DAPI (blue) is shown in the upper panel. Merged images (lower panel) show p24 in green (third line in gray), *S*. Typhimurium in red (second line in gray), and DAPI in blue. Z stacks of wide field fluorescent images were acquired, deconvoluted, and treated with ImageJ. Bar, 10 µm. Right panel—Intracellular bacteria may take advantage of arrested phagosome maturation in HIV-infected host cells, either **(A)** indirectly due to changes in intracellular trafficking or gene expression programs or **(B)** directly, through intracellular connections between *Salmonella*-containing vacuoles (SCV) and virus-containing compartment (VCC) that have neutral pH. The cell activation pathways and cytokine production were reported to be different between ST19 and ST313, although gene expression profiles are yet to be sufficiently dissected in the context of an HIV-infected host macrophage **(C)**. The humoral immune responses are biased, and anti-LPS antibodies are produced that are non-neutralizing and do not appear to block entry of bacteria **(D)**.

Non-typhoidal salmonellae have been identified early as HIV-related pathogens in both adults and children in sub-Saharan Africa. Although HIV and *Salmonella* coinfections have been the focus of several studies, most of these aimed to characterize the intrinsic properties of the invasive *Salmonella* compared with reference strains, rather than study the co-evolution of the bacteria with the HIV epidemy. Some reports have pointed to the dysregulation of inflammation induced by HIV. During non-typhoid *Salmonella* and HIV coinfection, human blood displays attenuated NFkB-mediated inflammation (127) (Figure 3). Cytokine changes in acute iNTS disease are correlated with cytokine signatures associated with macrophage functions and with sepsis. HIV–*Salmonella* coinfection had no major impact on the blood cytokines of patients (128). In one study, primary human alveolar macrophages from a small number of HIV-infected adults did not display any differential internalization and killing of bacteria but showed a dysregulation of cytokine responses to *Salmonella*. Increased quantities of TNF, IL10, and IL12 were released in the HIV-positive samples in response to the bacterial challenge. This may underlie the susceptibility to severe salmonellosis of patients with AIDS (129). It is worth noting, however, that cytokine read-outs might represent the “tip of the iceberg” for other profoundly modified cell phenotypes that have recently received new attention, like imbalanced metabolic status, cell death, or survival pathway. All of these pathways should be further studied by global analysis of gene expression profiles in infected and coinfected cells.

The intracellular relationship between the VCC and the SCV could be also studied with high-resolution microscopy to determine if there are structural connections between the two types of compartments and to ascertain whether one strain of bacteria is better at exploiting the mechanisms described in Figure 1. For example (and as cited above), the analysis of phagocytic function in HIV-*Salmonella* coinfected individuals could be aided by novel assays such as the detection of defective superoxide burst by flow cytometry on whole blood samples, as for HIV/tuberculosis-infected individuals (67).

Importantly, the dysregulated humoral immunity in HIV-infected individuals is characterized by high titers of inhibitory antibodies against *Salmonella* LPS. This is associated with defective killing of the bacteria, which relies more on antibodies against the outer membrane proteins (130). Therefore, the HIV infection prevents efficient humoral immunity without blocking bacteria from invading host cells. The bacteria can then remain hidden within host cells to disseminate throughout the body (Figure 3).

## Conclusion

The initial observation that macrophages are permissive to HIV infection was reported as early as in 1986. However, the effects of the viral infection on those phagocytic functions of macrophages and the emergence of opportunistic pathogens have been somewhat overlooked. One reason for that is that the reciprocal impact of the virus and the opportunistic pathogens on their common host is difficult to assess experimentally. Studying the cell biology of the *Salmonella* vacuole in HIV-infected hosts using tools developed recently for host–pathogen analysis, such as flow cytometry or advanced microscopy, may provide valuable information. In addition, the recent development of gene expression analysis, of both the bacteria and the host, in conjunction with single-cell analytical approaches, offers unprecedented opportunities for future studies to generate better understanding of the unique relationship between iNTS and the HIV-infected host. A better understanding of the interplay between HIV and these bacteria will have implications not only for treatment and management of *Salmonella* but also of other opportunistic pathogens.

## Author Contributions

Both authors have made a substantial, direct, and intellectual contribution to the work and approved it for publication.

## Conflict of Interest Statement

The authors declare that the research was conducted in the absence of any commercial or financial relationships that could be construed as a potential conflict of interest.
